# *Simulium reptans* (Linnaeus, 1758) and *Simulium reptantoides* Carlsson, 1962 from the Balkan Peninsula

**DOI:** 10.3897/zookeys.922.49306

**Published:** 2020-03-25

**Authors:** Jelena Đuknić, Vladimir M. Jovanović, Jelena Čanak Atlagić, Stefan Andjus, Momir Paunović, Ivana Živić, Nataša Popović

**Affiliations:** 1 Department of Hydroecology and Water Protection, Institute for Biological Research “Siniša Stanković” – National Institute of the Republic of Serbia, University of Belgrade, Bulevar despota Stefana 142, 11060 Belgrade, Serbia; 2 Faculty of Biology, University of Belgrade, Studentski Trg 16, 11000 Belgrade, Serbia; 3 Bioinformatics Solution Center, Freie Universität Berlin, Berlin, Germany

**Keywords:** genetic variability, Simuliidae, southeast Europe

## Abstract

*Simulium reptans* (Linnaeus, 1758) and *Simulium reptantoides* Carlsson, 1962 are two species of the *Simulium reptans* group whose distribution is unclear because of their confusing taxonomy and systematics. Their genetic variability is well known for populations in northern and central Europe and shows that both species have two forms; however, the genetic variability of these species in southern and eastern Europe is unknown. To identify the status of these two species in southeast Europe, mtDNA was extracted from 19 individuals from 12 localities across the Balkan Peninsula. Phylogenetic analysis confirmed the existence of two species with 7.38–7.94% divergence. Each species was comprised of two clades, with 2.31% and 1.43% interclade divergence for *S. reptans* and *S. reptantoides*, respectively. This study revealed the presence of both species across the Balkans and that *S. reptans* occurs in this area in only one form (*S. reptans* B), while *S. reptantoides* is found in two genetic forms (A and B).

## Introduction

The systematics, population genetics, distribution and evolution of black flies (Diptera: Simuliidae) represent interesting research fields for scientists worldwide (e.g., Hernández-Triana et al. 2012; Ya’cob et al. 2016; Ivković et al. 2016; Conflitti et al. 2017; Ruiz-Arrondo et al. 2018; Adler 2019). The great morphological similarity among certain species leads to frequent misidentification; nowadays, the description of new taxa is aided by cytogenetic and molecular identification methods. These methods have shown that some morphologically defined taxa consist of several sister species, which are usually reproductively isolated (Rothfels 1979; Adler et al. 2010). A similar situation occurs within the *Simulium reptans* group, which contains 16 species widely present in Europe and the Caucasus area (Adler 2019). The whole group consists of mammophilous and anthropophilous species, placing them in the focus of interest primarily because of their medical, sanitary and economic significance (Day et al. 2008; Kúdela et al. 2014).

Two species of this group, *Simulium reptans* (Linnaeus, 1758) and *Simulium reptantoides* Carlsson, 1962, have been extensively discussed in the literature. One of the problems with these species has been their frequent misidentification. Taxonomic features that distinguish them are given in only a few identification keys or scientific articles (Edwards 1920; Knoz 1965; Jedlička et al. 2004), while most of the keys did not include both species, which has led to inaccurate reports of their presence. According to Day et al. (2008), two main features could morphologically distinguish these two species: pigmentation of the cephalic apotome of larvae (Edwards 1920) and microtubercles on the thorax of pupae (Day et al. 2008). Likewise, *S. reptans* has a large and conspicuous dark spot in the middle of the cephalic apotome, while *S. reptantoides* has very little pigmentation except along the posterior margin. On the other hand, the pupae of these species can be distinguished by the average number of microtubercles on the thorax. Both species have two types of microtubercles (pointed and rounded), but their density on the thorax in *S. reptantoides* exceeds that in *S. reptans* pupae. Day et al. (2008) applied barcoding to individuals that were previously identified based on these morphological features, confirming that they could be distinguished by them.

The second problem regarding these species has been their nomenclature, which is reflected by the high number of synonyms (Adler 2019). Hence, *S. reptans* was previously described as *S. galeratum* (Edwards, 1920) (Crosskey and Howard 2004; Day et al. 2008; Bernotienė and Stunžėnas 2009), and *S. reptantoides* as *S. reptans* (Jedlička 1965; Day et al. 2008; Bernotienė and Stunžėnas 2009). The latest revision by Kúdela et al. (2014) described in detail the taxonomic and nomenclatural status of *S. reptans* and *S. reptantoides*. The recall of *S. reptantoides* from synonymy by Kúdela et al. (2014) was accepted by Adler and Crosskey (2014) in their annual inventory of world Simuliidae. In the present study we used the taxonomical approach of Kúdela et al. (2014), which was also adopted in the current inventory list (Adler 2019).

Both Day et al. (2008) and Kúdela et al. (2014) reported the existence of two different forms among *S. reptantoides*, termed A and B. The molecular diagnosis given by Day et al. (2008) was limited to British populations. Further examination by Kúdela et al. (2014) included European mainland populations as well. According to Kúdela et al. (2014), *S. reptantoides* is not found in the Baltic area and is limited to the UK and central Europe. *Simulium reptans* has a wider distribution (Scandinavia, UK, the Baltic area and Slovakia) and can also be found in its own forms, also named A and B (Kúdela et al. 2014).

According to the last inventory list (Adler 2019), *S. reptans* has a wide distribution and is present in south and eastern Europe, including the Balkan Peninsula, while *S. reptantoides* is limited to the UK and Slovakia.

Because of the work of Day et al. (2008) and Kúdela et al. (2014), the genetic variability of *S. reptans* and *S. reptantoides* is established for northern and central European populations. However, there are no data about the genetic variability of these species in southern and eastern Europe, even though *S. reptans* was frequently found in Balkan rivers (Crosskey 1998; Jedlička and Seitz 2008; Ivković et al. 2016). To the best to our knowledge, there are only a few published findings of *S. reptantoides* from the Balkans (Jedlička and Seitz 2008; Ivković et al. 2016).

The aim of the present study was to fill in the knowledge gap in the distribution and genetic variation of these two species in southeastern Europe, i.e., to determine whether they are present in the Balkans or not and if so, in which molecular form(s).

## Materials and methods

### Sample collection

**Figure 1.**
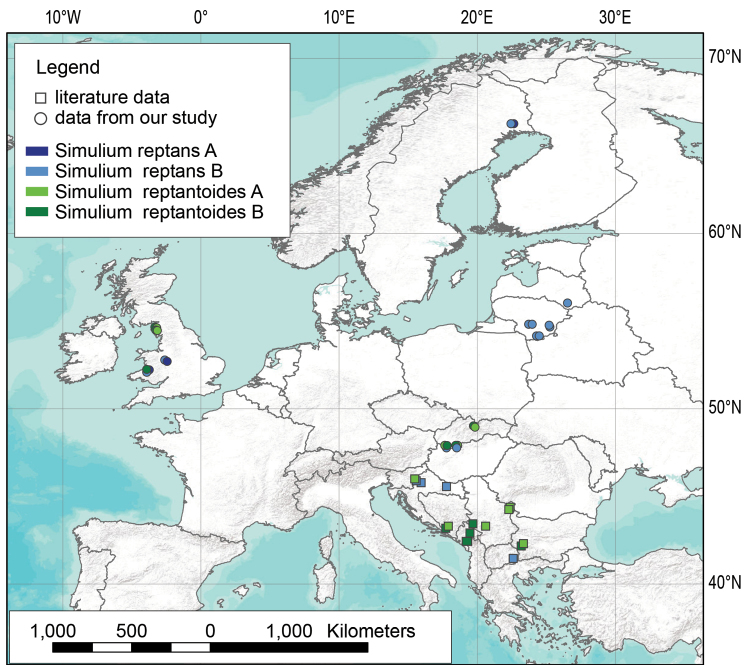
Map of the different localities. Localities of the collected specimen of *S. reptans* and *S. reptantoides* from the Balkan Peninsula (our study) and localities of the origin for the downloaded sequences from NCBI GenBank (literature data).

From 2015 to 2017, samples of larvae and pupae of *S. reptans* and *S. reptantoides* were collected at 12 localities across the Balkan Peninsula as follows: Slovenia (SVN), Croatia (CRO), Bosnia and Herzegovina (BIH), Montenegro (MNE), Serbia (SRB), North Macedonia (MKD) and Bulgaria (BGR) (Table 1 and Fig. 1). The collected material was preserved in the field in 96% ethanol. Identification was performed twice in the Institute for Biological Research “Siniša Stanković”. The material was identified before the molecular analyses of the specimens using the Rivosecchi (1978) and Lechthaler and Car (2005) identification keys, and once more after the molecular analyses using the identification keys and scientific articles as guidelines of Edwards (1920), Knoz (1965) and Day et al. (2008).

**Table 1.** Data for species *S. reptans* and *S. reptantoides* collected in the period 2015–2017. Species names are given according to the results of the study.

Accession Numbers	Species	Stage	River	Location	Country Alpha-3 code	Latitude /Longitude	Collection date	Collector
MK936587	*Simulium reptans*	pupa	Sava River	near Čatež	SVN	45.884078, 15.640831	03 Sep. 2015	Paunović et al.
MK936590		pupa	Sava River	near Čatež	SVN	45.884078, 15.640831	03 Sep. 2015	Paunović et al.
MK936588		pupa	Sava River	near Zagreb	CRO	45.759639, 16.047861	04 Sep. 2015	Paunović et al.
MK936589		pupa	Humljani	Humljani	CRO	45.578080, 17.798738	25 Sep. 2016	Đuknić et al.
MK947040		pupa	Strumica River	near Vasilevo	MKD	41.497500, 22.643333	24 Jun. 2017	Đuknić et al.
MK936596	*Simulium reptantoides*	larva	Sava River	near Čatež	SVN	45.884078, 15.640831	03 Sep. 2015	Paunović et al.
MK947041		pupa	Neretva River	near Počitelj	BIH	43.149052, 17.737837	31 Jul. 2016	Đuknić et al.
MK936595		larva	Zamna River	near Negotin	SRB	44.297883, 22.354969	26 Apr. 2015	Đuknić et al.
MK947046		pupa	Urovica River	near Urovica	SRB	44.399425, 22.407786	25 Apr. 2015	Đuknić et al.
MK947048		pupa	Urovica River	near Urovica	SRB	44.399425, 22.407786	25 Apr. 2015	Đuknić et al.
MK947045		pupa	Ibar River	near Raška	SRB	43.286957, 20.618514	11 Jun. 2017	Đuknić et al.
MK947047		pupa	Ibar River	near Raška	SRB	43.286957, 20.618514	11 Jun. 2017	Đuknić et al.
MK936591		pupa	Rila River	Rila	BGR	42.131866, 23.156651	17 Sep. 2017	Đuknić et al.
MK947044		larva	Neretva River	near Počitelj	BIH	43.149052, 17.737837	31 Jul. 2016	Đuknić et al.
MK947043		pupa	Neretva River	near Počitelj	BIH	43.149052, 17.737837	31 Jul. 2016	Đuknić et al.
MK946294		pupa	Tara River	near Kolašin	MNE	42.863386, 19.527027	08 Aug. 2017	Đuknić et al.
MK947042		pupa	Cjevna River	near Podgorica	MNE	42.382999, 19.278886	25 Mar. 2017	Đuknić et al.
MK940493		pupa	Lim River	near Prepolje	SRB	43.393293, 19.642978	09 Aug. 2016	Đuknić et al.
MK937592		pupa	Rila River	Rila	BGR	42.131866, 23.156651	17 Sep. 2017	Đuknić et al.

### Molecular procedures

DNA extractions from larvae and pupae were performed in the Institute for Biological research “Siniša Stanković”. To avoid the risk of contamination by other DNA sources, the intestinal tracts of the larvae were removed. For the extractions we used the isolation kit “KAPA2G Express Extract Kit” (Kapa Biosystems, United States, Wilmington, Massachusetts). The quality of the DNA was checked by agarose gel (1%) electrophoresis. The barcoding region of the mitochondrial COI gene of two morphologically identified species, *Simulium reptans* (five individuals) and *S. reptantoides* (14 individuals), was amplified using the following primers: LCO1490 (5-GGTCAACAAATCATAAAGATATTGG-3) and HCO2198 (5-TAAACTTCAGGCTGACCAAAAAAT CA-3) (Folmer et al. 1994). The total volume of mtDNA amplification was 25 μL, which contained 1 μL of extracted DNA, 16.9 μL of dH_2_O, 0.5 μL dNTPs, 0.5 μL GoTaq buffer, 0.7 μL of both primers and 0.2 μL of GoTaq polymerase. The PCR cycles were as follows: 2 min of denaturation at 95 °C, followed by 35 cycles of denaturation at 94 °C for 1 min, primer annealing at 50 °C for 1 min and extension at 72 °C for 1 min, the final extension step for 5 min at 72 °C. Ethidium bromide was used to visualise the PCR products on 1% agarose gels. DNA sequencing was performed at the Faculty of Biology, University of Belgrade (Center for Human Molecular Genetics). ABI Sequence Scanner Software v. 2.0 was used to check and arrange the sequences (Applied Biosystems). All DNA sequences were stored at GenBank; accession numbers are shown in Table 1.

### Genetic and phylogenetic analyses

In total, 90 sequences were analysed: five sequences of *S. reptans* and 14 of *S. reptantoides* collected from the Balkan Peninsula, 38 sequences of *S. reptans* and 33 of *S. reptantoides* downloaded from GenBank, and six sequences from the GenBank database were used as outgroups: two *Simulium vernum* Macquart, 1826, two *Thaumalea testacea* Ruthe, 1831 and two *Culicoides brevitarsis* Kieffer. The COI gene sequences for *S. reptans* and *S. reptantoides* that were downloaded from GenBank originated from Slovakia (19 sequences), Lithuania (8), Latvia (3), Sweden (12) and the UK (29), and are listed in Suppl. material 1: Table S1. MEGA6 (Tamura et al. 2013) with the ClustalW algorithm was used to align the sequences. The best-fitting model of sequence evolution was found in MEGA6 according to the model comparison procedure by the Bayesian information criterion (BIC) and log-likelihood (lnL) and was used in subsequent analyses.

Maximum likelihood (ML) and maximum parsimony (MP) phylogenetic analyses were also carried out using MEGA6 software (Tamura et al. 2013), while Bayesian phylogenetic analyses were performed using BEAST v2.4.2 (Bouckaert et al. 2014).

To assess branch support in the resulting ML and MP trees, 1,000 bootstrap replicates were performed. To calculate average genetic distances between clades and within each clade (bootstrap method: 1,000 replicates), the best-fitting model of base substitution was applied in MEGA6.

The best-fitting site evolution model priors within BEAST were selected according to a model selection run in MEGA6. We ran preliminary tests to examine the performance of strict versus uncorrelated log-normal relaxed clock priors. These preliminary analyses consisted of two independent runs, each for 6,000,000 generations, with sampling every 1,000 generation. We examined posterior density histograms in TRACER v1.6 (Rambaut et al. 2014) and concluded that strict clock priors better suit our data, and subsequently used these clock priors to reconstruct Bayesian phylogeny.

DnaSP v6.10.01 was used (Rozas et al. 2017) for the analyses of nucleotide diversity and tests of neutrality for each clade. The following parameters were obtained: number of used sequences (n), number of haplotypes (h), number of segregating sites (S), haplotype diversity (Hd) with the standard deviation, nucleotide diversity (Pi) with the standard deviation, Tajima’s D statistic (Tajima 1989), and Fu’s Fs (Fu 1997). The networks of *S. reptans* and *S. reptantoides* haplotypes from DnaSP were drawn in Network v5.0.0.1. (Librado and Rozas 2009). To reduce the number of nodes in the networks, star contraction (Forster et al. 2001) of haplotypes was conducted. The median-joining algorithm (Bandelt et al. 1999) was preformed to calculate the network.

## Results

Using the Lechthaler and Car (2005) identification key for morphological taxonomic identification, all sampled specimens were identified as *S. reptans*. However, barcoding of these individuals revealed that two species (*S. reptans* and *S. reptantoides*) were present among the identified material. Identification was then repeated using keys by Edwards (1920), Knoz (1965) and Day et al. (2008). After this revision, both species were morphologically identified. In the analysed material from all 12 localities, *S. reptantoides* made up 73% of the specimens, and morphological and genetic identification coincided 100%.

All retrieved sequences had lengths ranging from 453 bp to 606 bp. The Tamura 3-parameter model with the gamma distribution of variation between the nucleotide positions (Tamura 1992) fitted our collection of samples the best, as it had the lowest BIC score (Table 2).

**Table 2.** Five nucleotide substitution models that best fit the input data.

Model		BIC	lnL
T92+G	Tamura 3-parameter	7364.018442	-2643.969915
T92+G+I	Tamura 3-parameter	7372.132269	-2642.563464
HKY+G	Hasegawa-Kishino-Yano	7373.999596	-2638.033763
HKY+G+I	Hasegawa-Kishino-Yano	7381.914365	-2636.527782
TN93+G	Tamura-Nei	7384.845828	-2637.993514

**Figure 2.**
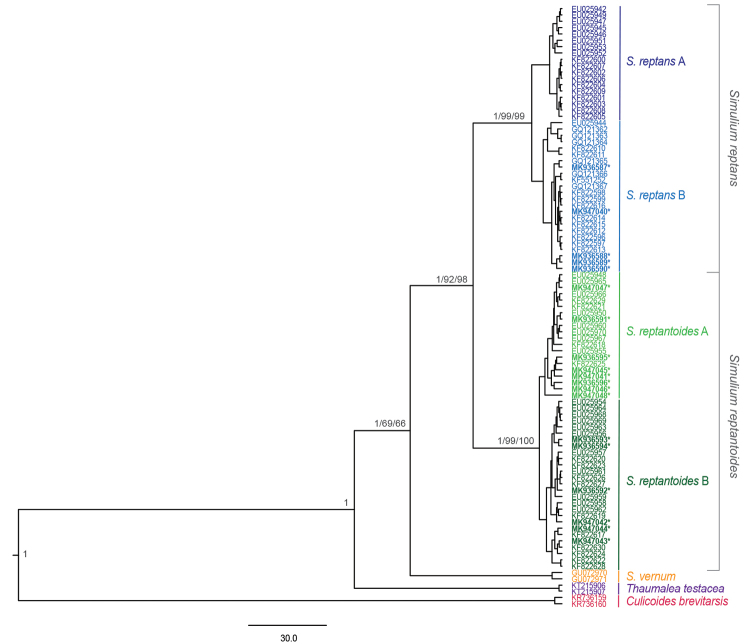
Bayesian phylogenetic tree based on the COI gene of two species, *S. reptans* and *S. reptantoides*. Species *S. vernum*, *Culicoides brevitarsis* and *Thaumalea testacea* were used as outgroups. Numbers above the branches represent posterior BA probabilities followed by ML and MP > 50% bootstrap support. Sequences (tree leaves) are given as GenBank accession numbers. Sequences in bold type with asterisks at the end of the accession number were obtained in this study. The colours of the clades are given according to the species and forms.

The topology of the phylogenetic tree for *S. reptans* and *S. reptantoides* involves seven clades (Fig. 2). The names for the clades (A and B) are given with respect to previous studies (Kúdela et al. 2014).

The Bayesian phylogenetic tree (Fig. 2) consisted of two highly supported monophyletic branches (with BI > 0.99) of *S. reptans* and *S. reptantoides*. One branch consisted of the clades *S. reptans* A and *S. reptans* B. The second branch consisted of *S. reptantoides* A and *S. reptantoides* B. Samples from the Balkan Peninsula occurred within three clades: *S. reptans* B, *S. reptantoides* A, and *S. reptantoides* B.

Nucleotide diversity within the monophyletic clades ranged from 0.50% within *S. reptantoides* B to 0.70% within *S. reptans* A (Table 3). The COI gene revealed a higher haplotype diversity (0.949) within the clade *S. reptantoides* B, while the lowest diversity (0.663) was detected within *S. reptans* B. The highest number (27) of haplotypes was also found in *S. reptantoides* B (Table 3). The negative values of Tajima’s D and Fu’s Fs (observed in all clades) indicate low nucleotide diversity but high haplotype diversity.

**Table 3.** Nucleotide diversity calculations and tests of neutrality; n – number of sequences, h – number of haplotypes, S – number of segregating sites, Hd – haplotype diversity ± standard deviation, Pi – nucleotide diversity ± standard deviation, Tajima’s D test and Fu’s Fs test.

Clades	n	h	S	Hd	Pi	Tajima’s D	Fu’s Fs
*S. reptans* A	18	9	20/453	0.797±0.090	0.00698±0.00252	-1.88682*	-1.912
*S. reptans* B	24	9	14/418	0.663±0.107	0.00667±0.00171	-1.07936	-1.485
*S. reptantoides* A	20	14	19/487	0.889±0.068	0.00631±0.00112	-1.72802	-8.315
*S. reptantoides* B	27	19	19/544	0.949±0.032	0.00500±0.00057	-1.79156	-16.054

Note: Statistical significance: *, p < 0.05

The interclade divergence for the COI sequence of *S. reptans* and *S. reptantoides* ranged from 1.43% (*S. reptantoides* A vs. *S. reptantoides* B) to 7.94% (*S. reptans* A vs. *S. reptantoides* A) (Table 4). Clades within species showed genetic distances that were 2.31% for *S. reptans* and 1.43% for *S. reptantoides* (Table 4).

**Table 4.** Evolutionary divergence between clades based on the pairwise analysis of COI sequences.

Clades	1.	2.	3.	4.
1. *Simulium reptans* A				
2. *Simulium reptans* B	0.0231			
3. *Simulium reptantoides* A	0.0794	0.0738		
4. *Simulium reptantoides* B	0.0775	0.0792	0.0143	

**Figure 3.**
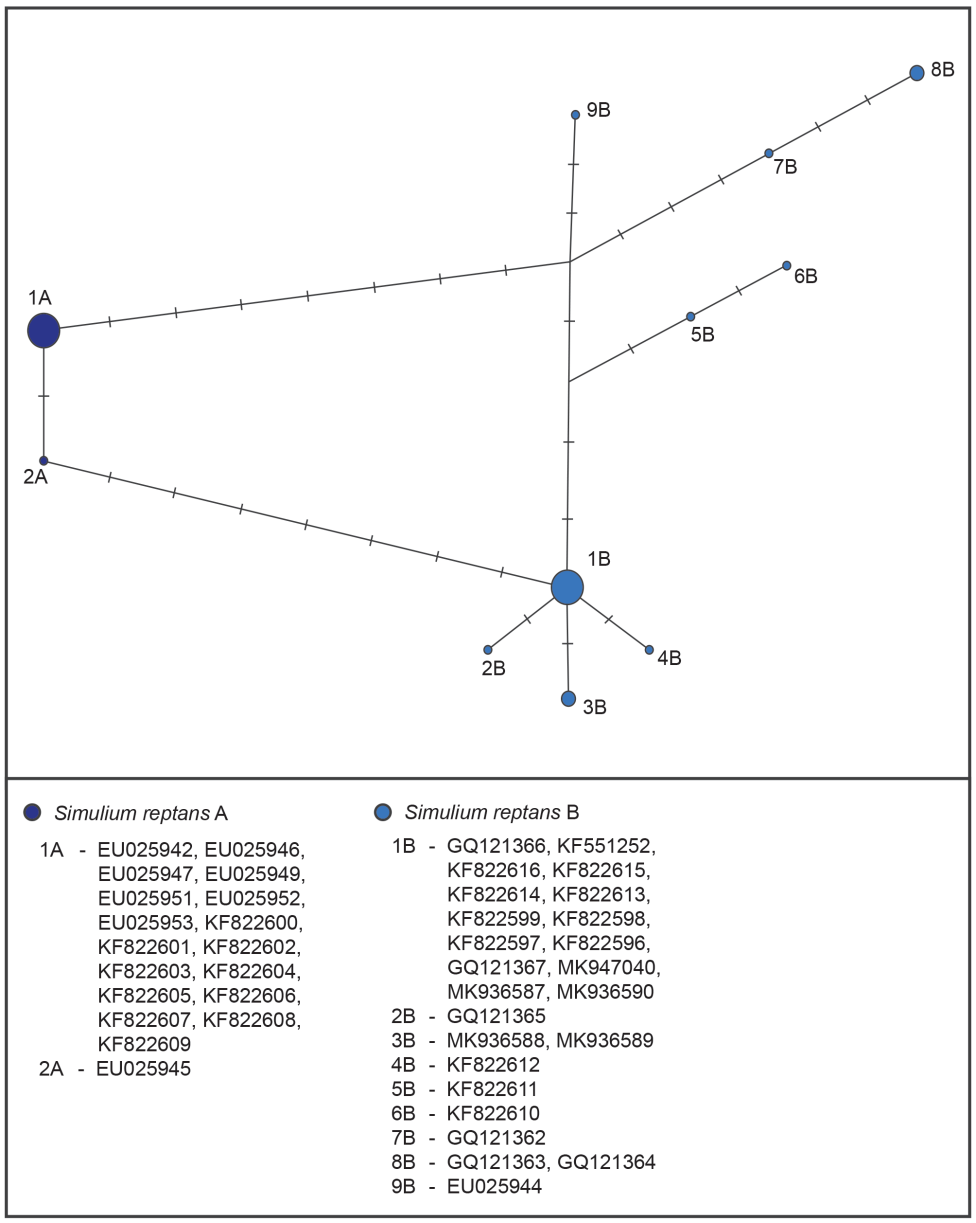
Haplotype network obtained from *S. reptans* mtCOI gene sequences using Network (Librado & Rozas, 2009). Circle sizes are proportional to the haplotype frequency. Colours and clade names correspond to the phylogenetic tree.

A total of 18 haplotypes of *S. reptans* were recognised in DnaSP (Table 3). After applying the star contraction method, the number of haplotypes was reduced to eleven. The minimum distance between haplotypes of *S. reptans* A and *S. reptans* B was seven mutation events. The overall lowest number of mutations (only one) was recorded between two haplotypes of the *S. reptans* A clade. All sequences were grouped in one haplotype except sequence number EU025945. The highest number of mutations in *S. reptans* B clade (nine) was found between haplotype 8B and haplotypes 2B, 3B, and 4B (Fig. 3).

**Figure 4.**
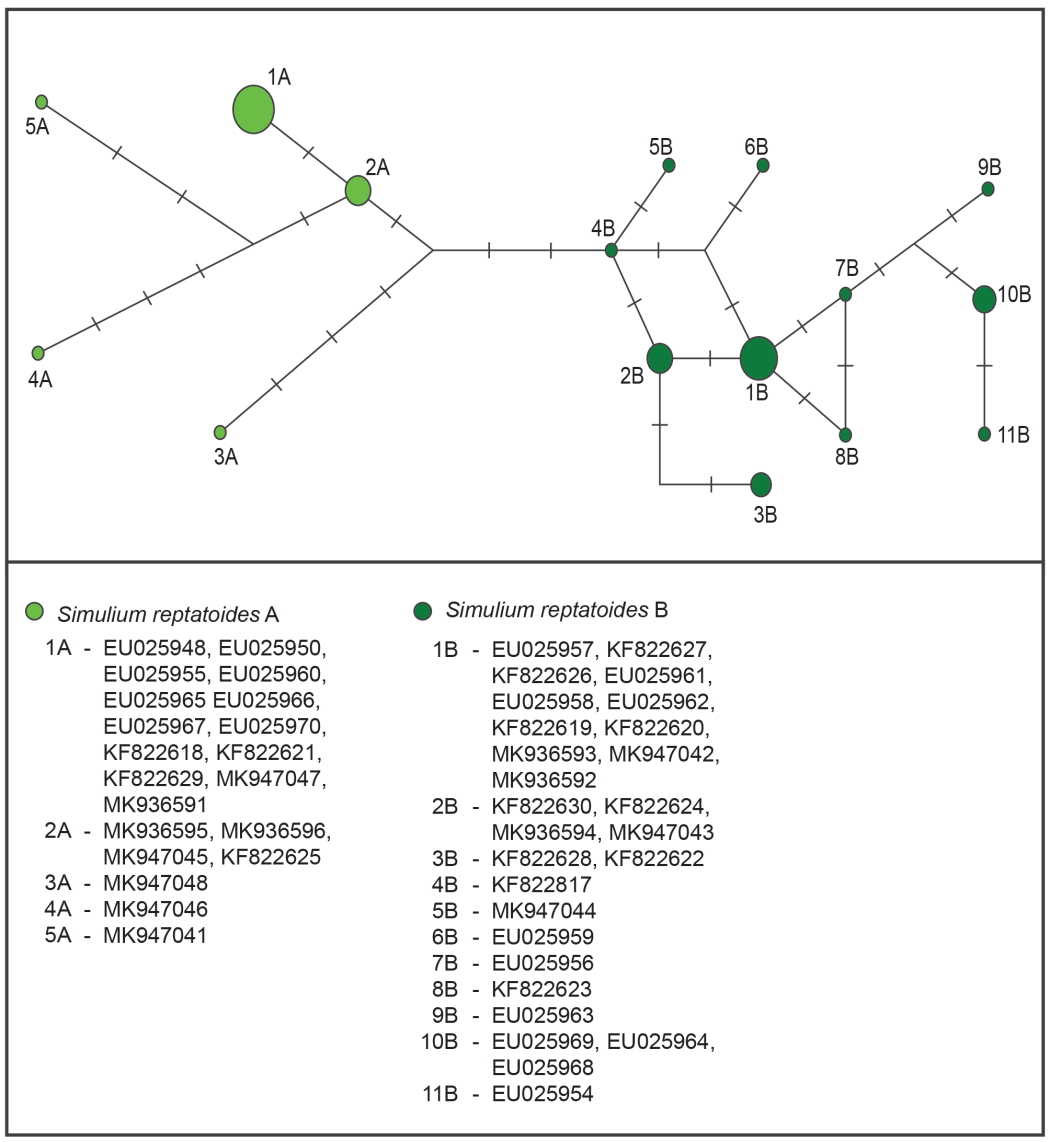
Haplotype network obtained from *S. reptantoides* mtCOI gene sequences using Network (Librado & Rozas, 2009). Circle sizes are proportional to haplotype frequency. Colours and clade names correspond to the phylogenetic tree.

A total of 33 haplotypes of *S. reptantoides* was recognised in DnaSP (Table 3). After applying the star contraction method, the number of haplotypes was reduced to 16 (Fig. 4). The minimum distance between haplotypes of *S. reptantoides* A and *S. reptantoides* B was three mutations. *Simulium reptantoides* A clade has five different haplotypes while the *S. reptantoides* B clade has 11.

## Discussion

Phylogenetic analyses of sequences from samples of the *Simulium reptans* group revealed the presence of two major branches with four well-distinguished clades. Two branches represent previously defined species, *S. reptans* and *S. reptantoides* (Edwards 1920; Knoz 1965; Day et al. 2008). The divergence between them (7.38–7.94%) confirmed the existence of two species. According to previous studies (Rivera and Currie 2009; Hernández-Triana et al. 2012; Đuknić et al. 2019), genetic divergences in the range of 2.83–15.33% suggest the existence of different species, while genetic divergences in the range of 0–3.84% suggest intraspecific differences.

The typology of trees using different methods (ML, MP and Bayesian) showed the same position of the main clades, with high bootstrap values. We explain above the Bayesian tree topology. The positions of some lineages within these clades differed among the ML, MP and Bayesian phylogenetic trees. However, these differences do not have high bootstrap support and need to be analysed further.

Each species consisted of two clades that represented different molecular forms, A and B. The existence of these forms was described by Day et al. (2008) and Kúdela et al. (2014), and no new forms were defined within samples from the Balkan Peninsula. The interclade divergences for the COI sequence of these two forms in *S. reptans* (2.31%) and *S. reptantoides* (1.43%) were insufficient to consider them as different species. However, these percentages suggest a high intraspecific variability in both species. The high variability could be related to wide distribution.

According to the latest inventory list, *S. reptans* is present in some Balkan countries, including Bosnia and Herzegovina, Greece, North Macedonia, Montenegro and Serbia. Kúdela et al. (2014) showed that the *S. reptans* A form occurs only in the UK and Sweden, while *S. reptans* B, although it is present in the UK and Sweden as well, albeit with infrequent findings, is mainly distributed in central Europe and the Baltic area. Our results revealed the presence of the *S. reptans* B form in the Balkans as well. One haplotype (3B) was found exclusively in Balkan samples (Croatia), while another (1B) was found in both Balkan samples (Slovenia and North Macedonia) and in Slovakia and Lithuania. We confirmed the low variability in the *S. reptans* A form (with only two haplotypes present and only one mutational step difference between them) and its restricted distribution in western and northern Europe. According to our results and with the inclusion of all the samples from the Balkan Peninsula, the *S. reptans* B form demonstrated a wider distribution than was previously known.

*Simulium reptantoides* was originally described by Carlsson from an unspecified European country; thus, its type locality is unknown (Adler 2019). The species was subsequently confirmed from Britain and Slovakia (Kúdela et al. 2014). Although some rare and sporadic findings of *S. reptantoides* exist, they are mostly limited to the northern Balkan area, the Danube and the Sava rivers in Croatia (Ivković et al. 2016) and the Danube drainage system (Jedlička and Seitz 2008). In the study of Kúdela et al. (2014), *S. reptantoides* was limited to the UK (predominantly the A form) and central Europe (predominantly the B form). Our research showed a uniform distribution of both forms throughout Europe, from the UK, through Slovakia, to the Balkan Peninsula (Slovenia, Serbia, Bosnia and Herzegovina, Montenegro and Bulgaria). Both forms were found at the same sampling site, overlapping at all life stages. Furthermore, haplotype diversity was higher than the one observed in *S. reptans*. The samples collected from the Balkan Peninsula appeared as the most basal within the *S. reptantoides* A form, while being interspersed within the B form clade. This points to the importance of the Balkan Peninsula as a potential place of origin for clade A, but also as a place of high simuliid genetic diversity.

## Conclusions

With the use of molecular barcoding, this study confirmed the presence of *S. reptans* throughout the Balkans and revealed that *S. reptantoides* is more widely distributed and has a higher frequency of occurrence in the Balkans than *S. reptans*. Based on previous studies (Day et al. 2008; Bernotienė and Stunžėnas 2009; Kúdela et al. 2014), we established a wider distribution for both species. The genetic variation of *S. reptans* and *S. reptantoides* suggests the existence of different forms (A and B). This study showed that in the Balkans, only one form of *S. reptans* is present (form B), while *S. reptantoides* occurs in both forms (A and B).

The presence of *S. reptantoides* on the Balkan Peninsula indicates that some previous findings were misidentified or synonymised. Further analyses are needed in order to precisely delimit the distribution of this species and to explain the high intraspecific variability.

## Supplementary materials

Supplementary material 1 The COI gene sequences for *S. reptans* and *S. reptantoides* downloaded from GenBank Data type table Explanation note Sequence downloaded from GenBank, Author of the sequence and Country where the original material was collected. https://binary.pensoft.net/file/393891This dataset is made available under the Open Database License (http://opendatacommons.org/licenses/odbl/1.0/). The Open Database License (ODbL) is a license agreement intended to allow users to freely share, modify, and use this Dataset while maintaining this same freedom for others, provided that the original source and author(s) are credited.Jelena Đuknić, Vladimir M. Jovanović, Jelena Čanak Atlagić, Stefan Andjus, Momir Paunović, Ivana Živić, Nataša Popović
